# Computationally aided design of a high-performance organic semiconductor: the development of a universal crystal engineering core†
†Electronic supplementary information (ESI) available: Synthetic details, characterization of synthesized materials, absorption spectra, and additional information on computational details and device fabrication. CCDC 1833615–1833619 and 1922871–1922873. For ESI and crystallographic data in CIF or other electronic format see DOI: 10.1039/c9sc02930c


**DOI:** 10.1039/c9sc02930c

**Published:** 2019-10-07

**Authors:** Anthony J. Petty, Qianxiang Ai, Jeni C. Sorli, Hamna F. Haneef, Geoffrey E. Purdum, Alex Boehm, Devin B. Granger, Kaichen Gu, Carla Patricia Lacerda Rubinger, Sean R. Parkin, Kenneth R. Graham, Oana D. Jurchescu, Yueh-Lin Loo, Chad Risko, John E. Anthony

**Affiliations:** a Department of Chemistry , University of Kentucky , Lexington , Kentucky 40506-0055 , USA . Email: anthony@uky.edu; b Department of Chemical and Biological Engineering , Princeton University , Princeton , New Jersey 08544 , USA; c Department of Physics and Center for Functional Materials , Wake Forest University , USA; d Physics and Chemistry Institute , Federal University of Itajubá , 37500-903 , Itajubá , MG , Brazil; e Andlinger Center for Energy and the Environment , Princeton University , Princeton , New Jersey 08544 , USA; f Center for Applied Energy Research , University of Kentucky , Lexington , Kentucky 40511 , USA

## Abstract

Herein, we describe the design and synthesis of a suite of molecules based on a benzodithiophene “universal crystal engineering core”. After computationally screening derivatives, a trialkylsilylethyne-based crystal engineering strategy was employed to tailor the crystal packing for use as the active material in an organic field-effect transistor. Electronic structure calculations were undertaken to reveal derivatives that exhibit exceptional potential for high-efficiency hole transport. The promising theoretical properties are reflected in the preliminary device results, with the computationally optimized material showing simple solution processing, enhanced stability, and a maximum hole mobility of 1.6 cm^2^ V^–1^ s^–1^.

## Abstract 2

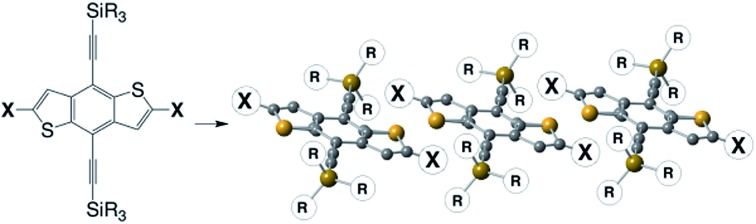
Silylethyne-functionalized benzodithiophene serves as a universal crystal engineering core to yield stable, soluble, π-stacked arrays of aromatic chromophores.

## Introduction

The solid-state arrangement of molecules in crystalline films is the determining parameter for the performance of organic semiconductors. While numerous crystal engineering paradigms exist for tuning molecules functionalized with a variety of supramolecular synthons^1^ for exploration of improved pharmaceuticals,^2^ tuning of solid-state reactivity,^3^ photochemistry,^4^,^5^ and crystal mechanical properties,^6^ the crystalline order of the simple nonpolar aromatic molecules used as semiconductors is typically more difficult to control. Apart from alignment induced by the van der Waals interactions of long hydrocarbon chains (the so-called “zipper” or “fastener” effect),^7^ few reliable approaches exist that can improve solubility and control solid-state order in simple aromatic structures. Nearly twenty years ago, we introduced silylethyne substitution as a simple method to increase stability, improve solubility, and induce π-stacking in aromatic molecules.^8^,^9^ This functionalization strategy has been applied to acenes,^10^–^16^ heteroacenes,^17^–^25^ indenofluorenes,^26^ benzo-thiadiazoles,^27^ zethrene,^28^ and others,^29^–^32^ typically *via* a simple alkynylation sequence,^33^ to yield new materials for transistor,^34^–^38^ photovoltaic,^39^–^44^ imaging,^45^ light-emitting,^46^,^47^ and other applications.^48^–^50^ While this approach is relatively general in scope, it cannot be applied to a number of promising chromophores due to difficulties in preparing the necessary precursors, or to the delicacy of certain chromophores that preclude ethynylation.

In order to both apply silylethyne functionality to a more general class of materials and, at the same time, integrate computation-based guidance to assess a derivative's likelihood for high performance in an application, we envisioned developing a “universal crystal engineering core” that could be orthogonally derivatized to allow manipulation of the properties of the aromatic backbone while still exploiting the versatility of silylethyne-based crystal engineering strategies to modify the solid-state ordering. Through simple functionalization strategies our universal crystal engineering core could be used, along with feedback from computation, to develop high-performance materials. The design parameters we required for a universal crystal-engineering core are straightforward – simple and scalable preparation, amenable to addition of functionalized alkynes to tune crystal packing, and halogen “handles” to allow attachment of the chromophores of interest. Further, silylethyne-based crystal engineering typically works best with roughly linear molecules, which suggests that the core should terminate in five-membered rings; as such, a benzodithiophene (BDT) core appeared to be an ideal scaffold. We report here our first example of our modular crystal engineering approach to new organic materials; the development of stable, new molecules for organic field-effect transistors (OFETs).

## Results and discussion

### Design and synthesis of universal crystal engineering core

**Fig. 1:**
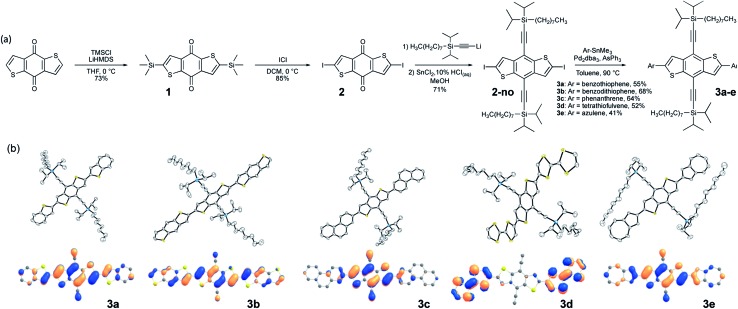
(a) Scheme showing the synthetic route to **3a–e**. (b) Top row: thermal ellipsoid plots of structures of derivatives **3a–e**, showing the overall planar configuration of the core obtained in all derivatives. Bottom row: pictorial representations of the HOMO of **3a–e** where the degree of HOMO delocalization is influenced by the pendant group. The side chains are trimmed down to alkynyl groups in the calculations and hydrogens are omitted to enhance clarity.

Our approach begins with the development of a multi-gram synthetic approach to diiodo-benzodithiophene quinone, 2, through a novel, scalable method that obviates the need for cryogenic temperatures (Fig. 1a).^51^ The crystal engineering silylethyne handle is added by routine ethynylation/deoxygenation sequence to provide the desired core.

The size of the trialkylsilyl group can be modified depending on the combined size of core and pendant; our previous work produced a model for controlling solid-state order that relates the nature of π-stacking to the diameter of the solubilizing group relative to the length of the chromophore.^10^ Since even small chromophores appended to this core will yield molecules with substantial aspect ratio, commercially available trialkylsilyl groups are unlikely to yield appropriate π-stacking motifs. For our initial studies, we employed the *n*-octyldiisopropylsilyl derivative as the trialkylsilyl group, which has shown promise in large aromatic systems such as the bistetracenes.^30^

### Screening of aromatic pendants

We employed modified (Farina) Stille coupling conditions^52^,^53^ to attach the pendant to the crystal engineering core, first appending benzothiophene and BDT to **2-no**, giving products **3a** and **3b**. Yields were generally good after minimal optimization, producing stable, soluble materials that were purified by standard chromatographic techniques. Crystals suitable for X-ray analysis were easily grown from slow cooling of saturated solutions, and the diffraction measurements confirmed the planarity of the chromophores and π-stacked order of the materials, consistent with previous reports on bithiophene linkages.^54^ To determine whether linking 6-membered aromatic rings to core **2-no** would also yield the planar systems required for efficient π-stacking, we appended phenanthrene to **2-no** to give **3c**. Crystallographic analysis showed a planar molecule, with strong π-stacking interactions and significant intermolecular overlap. Finally, applying this approach to some of the more delicate chromophores of interest to the organic electronics community, we coupled both tetrathiafulvalene (TTF)^55^ and azulene to **2-no**. Here again, the products were stable, soluble, crystalline materials with planar backbones and strongly π-stacked arrangements in the solid state. All structures have been submitted to the Cambridge Crystallographic Data Centre – registry numbers can be found in the ESI.†

The photophysical properties of the chromophore-core-chromophore systems are determined by the appended groups (Fig. S1†). When there is little donor–acceptor character between the core and pendant groups (benzothiophene, benzodithiophene, phenanthrene) minimal absorption shifts are observed. The azulene-pendant compound **3e**, which has a highest-occupied molecular orbital (HOMO) that is predominately localized on the BDT core (Fig. 1b, bottom row), caused a substantial red shift in absorption (the LUMOs were similarly localized, see Fig. S11†). Similarly, the strongly red-shifted and relatively featureless absorption for the TTF derivative **3d** suggests charge-transfer between the TTF unit and the core, with the HOMO localized to the pendants and the LUMO localized to the core.

To gain a measure of the difference in stability between the materials prepared from our benzodithiophene core compared to a prototypical organic semiconductor, tri-isopropylsilylethynyl pentacene (TIPS-pentacene), the decay observed in the solution absorption spectra of **3b** and TIPS-pentacene were followed under intense external lighting as a function of time (Fig. S2†). While the TIPS-pentacene absorption declined rapidly, with a half-life of less than 15 minutes, **3b** exhibited no appreciable decomposition after 7 hours under the same conditions. A high degree of solution stability is important for development of viable semiconductor inks.

With the exception of the TTF derivative **3d**, all synthesized derivatives adopt extended π-stacked arrangements (Fig. S3†) in the 1-D slipped-stack motif, with reasonably close contacts between the stacks. In our prior studies on acenes, such packing arrangements typically arose in systems where the solubilizing trialkylsilyl groups were not sufficiently large to induce the 2-D “brickwork” packing motif that is more desirable for charge tranpsort.^10^ In contrast, **3d** adopted an end-to-end arrangement that yielded a chevron pattern similar to that seen in the orthorhombic polymorph of rubrene (Fig. S3†).^56^

### Computational selection of candidates for device evaluation

The frontier molecular orbitals (MO) of **3a–e** were evaluated by density functional theory (DFT) calculations at the ωB97X-D/Def2SVP level of theory after geometry optimization (Fig. 1b, bottom row).^57^,^58^ The intermolecular overlaps of the HOMOs in the solid state are critical for hole transport, thus for this application materials where this orbital spans the length of the molecule are preferred. We observe that the HOMOs are delocalized along the long axes of the chromophores to different degrees depending on the pendant groups (Fig. 1b, bottom row). The HOMOs of **3a** and **3b** are delocalized along the entire chromophore, whereas the HOMOs are localized on the central BDT core in **3c** and **3d**, and is localized on the pendant azulene moieties in **3e**. Thus, the BDT pendant **3b**, showing HOMO delocalization along the entire conjugated backbone, was selected for further crystal engineering efforts.^57^,^58^

### Crystal engineering to improve crystal packing

**Fig. 2:**
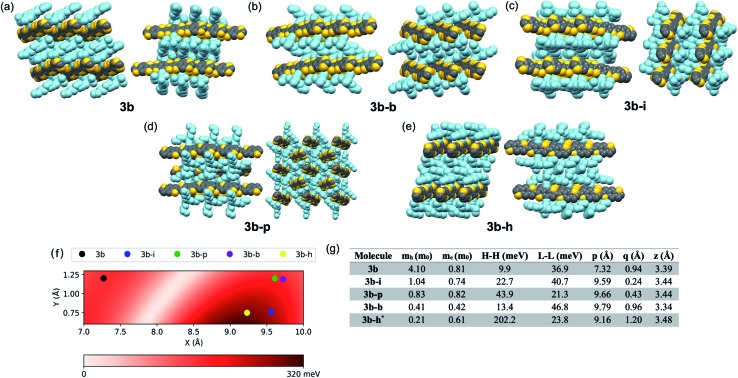
(a–e) Space-filling representation of the crystal packing of derivatives of **3b**, **3b–b**, **3b–i**, **3b–p**, and **3b–h**, in all cases showing a projection of the crystal structures, looking down the *a* axis on the left of the group and looking down the *b* axis on the right of the group, to assess the qualitative overlap of π-surfaces in the solid state. Sidechains are colored light blue for clarity. (f) Electronic couplings as a function of intermolecular slip in a dimer model. *X* and *Y* indicate long and short axis slip, respectively. (g) Effective masses for holes (*m*_h_) and electrons (*m*_e_) calculated at band extrema, along with largest HOMO–HOMO (H–H) and LUMO–LUMO (L–L) electronic couplings calculated from dimer models. The last three columns show the long-axis slip (p), short-axis slip (q), and vertical slip (z) for each dimer, respectively. For all crystals, the dimer with the largest H–H coupling is also the one with the largest L–L coupling. As the crystal structure of **3b–h** did not fully refine, computational models were built based only on the atomic positions of the backbone atoms.

While **3b** demonstrated good delocalization of the HOMO, its crystal packing was still not optimized for use in devices where charge transport is the key metric; the trialkylsilyl group required further tuning to optimize crystal packing for the desired application. Due to the large aspect ratio of the molecule, we focused on the synthesis of silyl derivatives containing relatively long alkyl groups, such as tri(iso-butyl)silyl (**3b–i**), tri(*n*-butyl)silyl (**3b–b**), tri(*n*-pentyl)silyl (**3b–p**), and tri(*n*-hexyl)silyl (**3b–h**). The syntheses simply required addition of the relevant alkynes to **2**, followed by coupling of the BDT pendant to the new cores, allowing rapid screening of solid-state order as a function of the size of the trialkylsilyl group. Single crystals were grown and analyzed for all five derivatives. Molecules **3b–b** and **3b–i** yielded a crystal packing that was in-between the 1D slip-stack and the 2D-brickwork π-stacking motifs,^10^ with both showing a clear co-facial overlap of π-surfaces in one direction (Fig. 2b and c, left), but with slight geometric overlap in the second direction (Fig. 2b and c, right). The crystal packing of **3b–p** showed insulated 1D π-stacks (Fig. 2d). However, **3b–h** exhibited exceptional overlap of the π-surfaces in both directions, clearly demonstrating the archetypal 2D-brickwork packing (Fig. 2e). Unfortunately, full structural refinement of crystals of **3b–h** was not possible due to extensive disorder of both the backbone and side chains, and as such the solved but unrefined structure is presented here strictly to demonstrate the overall packing motif.

**Fig. 3:**
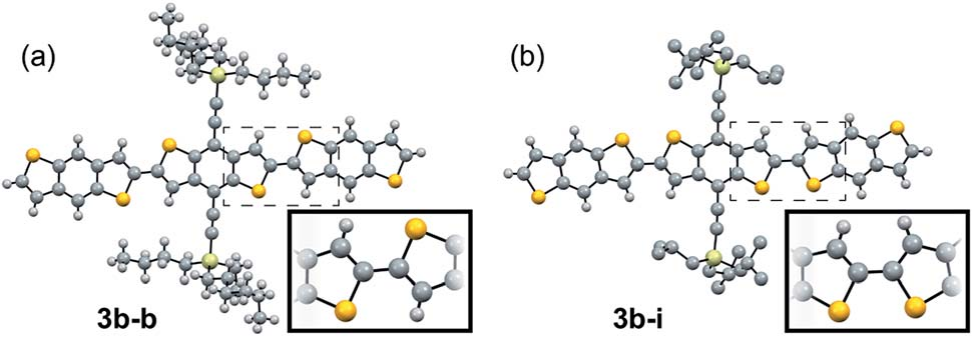
Ball and stick representation of the major conformers of **3b–b**, *anti*, (a) and **3b–i**, *syn*, (b) as determined from their respective crystal structures. Inset shows relevant atom positions.

Looking more closely at these crystal structures, careful assessment of the occupancies of the sulfur atoms of adjacent BDT units in **3b** derivatives revealed that many consist of a mixture of *anti* and *syn* conformers in adjacent BDT units. This disorder is unpredictable and varied between derivatives. **3b** showed an ∼80 : 20 *anti* : *syn* relationship whereas **3b–b** showed a 96 : 4 *anti* : *syn* relationship (Fig. 3a). **3b–i** was peculiar as its crystal structure revealed the adjacent BDT units exhibited a majority *syn* relationship (Fig. 3b). The presence of this conformational disorder may present challenges, as the mixture conformations has been related to decreased device performance due to increased disorder^59^ but could also lead to stronger electronic couplings.^60^ Unfortunately, with the present system it is not possible to deconvolute the effect that the rotational disorder may have on the material properties, nor to quantify the precise amount of rotational disorder in thin films of the materials. We will report our approach to mitigate this disorder issue in future work.

### Computational assessment of electronic couplings

Intermolecular electronic couplings among the frontier MOs were calculated at the ωB97X-D/Def2SVP level of theory based on dimers extracted from the molecular crystals. The largest electronic couplings are shown in Fig. 2f, along with the carrier effective masses calculated based on parabolic approximation from the electronic band structure determined by periodic DFT calculations, as shown in Fig. S10 and S11 (see ESI for computational details†). While the transport properties largely depend on the crystal packing arrangements, it is worth noting that intermolecular slip could have a non-trivial effect on electronic couplings – *i.e.*, electron–phonon couplings could play an important role in charge-carrier transport. To give a qualitative description of this effect, we calculated the HOMO–HOMO electronic coupling as a function of relative in-plane slip within a dimer model. After optimizing the geometry of the **3b** core where the side chains are trimmed down to alkynyl groups, we built the dimer model with a set of slips defined by the long and short molecular axes and the intermolecular axis of the dimer. The HOMO–HOMO electronic coupling is plotted against varying long/short axis slips while the intermolecular distance is fixed at 3.4 Å, as shown in Fig. 2f. From this analysis, it is clear that there are regions with very small or large electronic couplings, as expected.^60^,^61^ In our crystals, the displacements of **3b–h** put this derivative near the region featuring the largest electronic couplings. We note the effect of side chain trimming seems to be negligible as the difference in HOMO–HOMO electronic coupling between trimmed/untrimmed dimers of **3b–i** is calculated as less than 3 meV. On the other hand, the electronic couplings of **3b** derivatives extracted from Fig. 2f and g are generally larger than those from dimer models built from experimentally determined crystal structures, which implies the importance of nuances in atomic positions. In **3b–h**, the HOMO–HOMO coupling reaches more than 200 meV, and the hole effective mass is only 0.21*m*_0_, making it a prime candidate for device evaluation. While a small electronic coupling in the dimer model is typically related to heavy carriers, in **3b–b** the hole effective mass is rather small, suggesting some limitation in approximating the crystal electronic structure with isolated dimer models, and it too may be a strong candidate for device evaluation. Notably, the electronic couplings are sensitive to even sub-Å slips of these large backbones; for instance, the HOMO–HOMO couplings in **3b–b** and **3b–h** are quite different even though the slips are similar. This result points to the potential further limitation of evaluating these systems with such a static (*e.g.* single-point calculation) representation, and that non-local electron–phonon couplings could play an important role in these systems.^62^,^63^

### Device performance of computationally selected derivatives

Using the information gleaned from the electronic structure calculations, compounds **3b–b** and **3b–h** were selected and evaluated for their performance in organic field-effect transistors. Details on device fabrication can be found in the ESI.† Devices of **3b–b** and **3b–h** were fabricated using an aligned drop-cast method,^64^ and exhibited average hole mobilities of 0.15 ± 0.04 cm^2^ V^–1^ s^–1^ and 0.7 ± 0.3 cm^2^ V^–1^ s^–1^, with maximum mobilities of 0.21 cm^2^ V^–1^ s^–1^ and 1.52 cm^2^ V^–1^ s^–1^, respectively (Fig. S6†). Devices fabricated from **3b–h** also demonstrated low threshold voltages, (1 ± 5 V) and subthreshold swings of 1.5 ± 0.5 V dec^–1^.

**Fig. 4:**
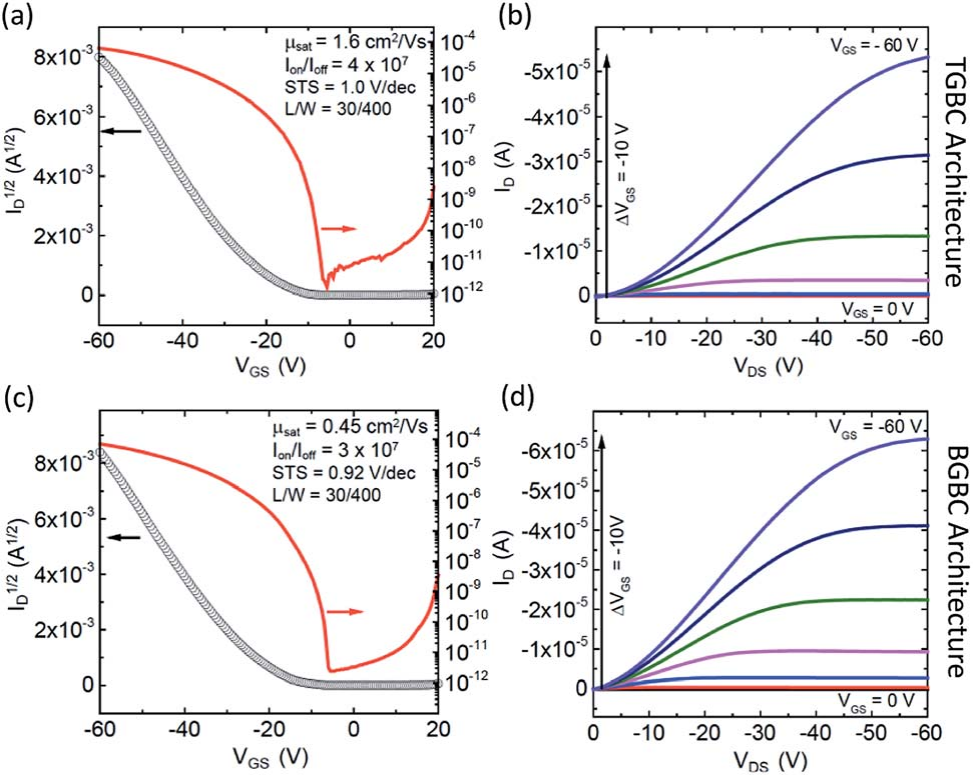
Current–voltage characteristics of the best performing device using **3b–h** as the semiconductor in the TGBC architecture (a and b) and the BGBC architecture (c and d). (a and c) Evolution of the drain current *I*_D_ as a function of gate-source voltage *V*_GS_ in the saturation regime at *V*_DS_ = –60 V. The left axis shows the square root of *I*_D_ while the right axis shows *I*_D_ in a log-scale. (b and d) Evolution of *I*_D_ as a function of drain-source voltage *V*_DS_ at different fixed values of *V*_GS_.

Since compound **3b–h** showed the most encouraging performance in OFETs, along with the highest calculated electronic couplings, we further explored performance in devices with different architectures. Bottom-gate, bottom contact (BGBC) OFETs were fabricated by spin coating the organic semiconductor over a Si/SiO_2_ substrate with pentafluoro benzenethiol (PFBT)-treated gold source and drain electrodes. Standard procedures were adopted to characterize and analyze these transistors.^65^ Measurements performed under N_2_ on 45 devices yielded a maximum mobility of 0.45 cm^2^ V^–1^ s^–1^ and an average of 0.23 ± 0.07 cm^2^ V^–1^ s^–1^ in this configuration. The mobility histogram and the current–voltage characteristics of the best performing device are included in Fig. S7†, 4c and d, respectively. These devices typically exhibit large on/off current ratios (10^6^–10^7^) and sharp turn on (subthreshold swings around 1 V dec^–1^), however the threshold voltage is relatively large and negative (–20 to –30 V) suggesting that this material interacts strongly with traps at the interface with the SiO_2_ dielectric.^66^ Since co-planar contacts typically produce large contact resistance and SiO_2_ creates significant scattering at the semiconductor/dielectric interface,^65^,^67^,^68^ we incorporated the same compound in OFETs with a top-gate bottom contact (TGBC) architecture,^69^ employing parylene as the top-gate dielectric. These OFETs provided mobilities as high as 1.6 cm^2^ V^–1^ s^–1^ (Fig. 4a and b) with an average of 0.7 ± 0.3 cm^2^ V^–1^ s^–1^ obtained on 34 devices.

The curvature of the current–voltage curves at low *V*_DS_ (Fig. 4b and d) present in both architectures is a typical signature of high contact resistance, suggesting that the mobility in these devices is limited by charge injection. Such injection barriers may be attributed to a number of different variables, including an energetic barrier to hole injection, local microstructural differences near the electrode that impede charge transport from the contact to the accumulation layer, dipoles formed at the semiconductor/electrode interface, or tunnelling resistance of the PFBT layer.^70^ To further assess this issue, we measured the ionization energies of both **3b–b** and **3b–h** using ultraviolet photoelectron spectroscopy, yielding values between 5.2 and 5.3 eV (Fig. S12 and S13†). Device performance could thus likely be further enhanced by improving the contacts.

### GIXD evaluation of thin-film structure

To fully characterize the structures accessed in thin-films of **3b–h**, and explore the possibility of thin-film polymorphism in these materials, grazing incidence X-ray diffraction (GIXD) was employed on both the aligned drop-cast films^64^ and spin-cast films. The as-cast films were also subjected to post-deposition thermal annealing (TA) and solvent vapor annealing (SVA)^71^ to assess polymorphic stability, as these techniques have demonstrated the ability to induce polymorphic transformations in organic small molecules.^72^–^74^

**Fig. 5:**
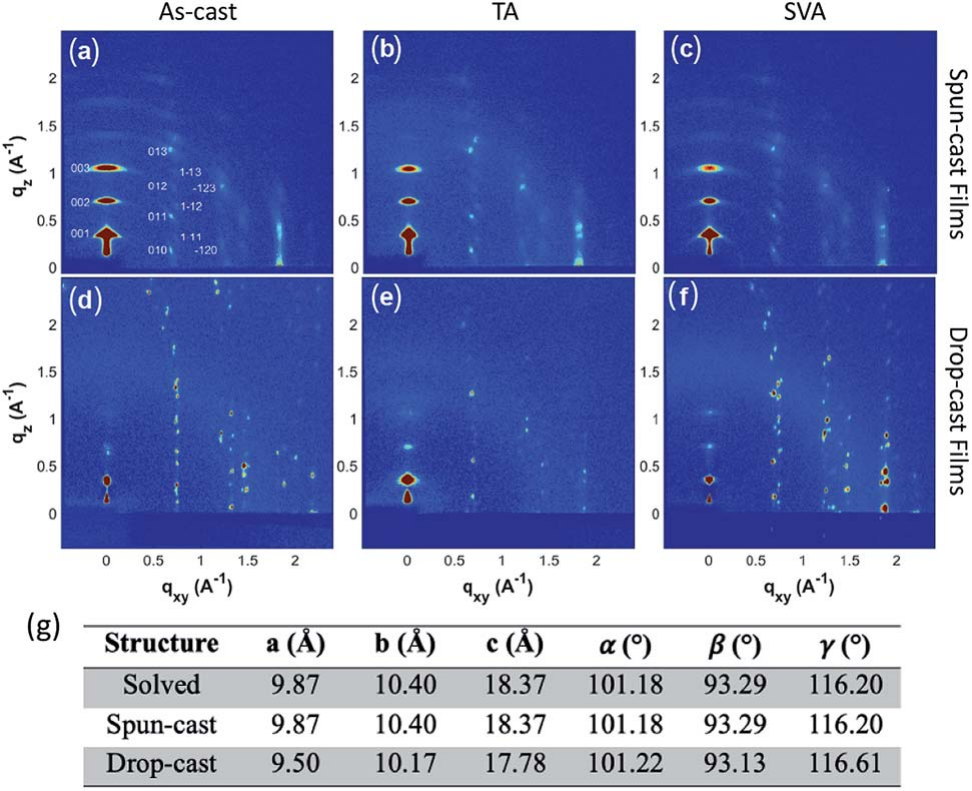
Grazing-incidence X-ray diffraction pattern of (a) a spun-cast thin film of **3b–h**. X-ray patterns after the spun-cast thin film is (b) TA and (c) SVA. X-ray patterns of (d) drop-cast **3b–h** and after the film had been (e) TA and (f) SVA, respectively. (g) Unit-cell parameters of the solved crystal structure along with those determined from the spun-cast and drop-cast films.

**3b–h** is crystalline as-deposited by both spin-casting (Fig. 5a) and aligned drop-casting (Fig. 5d). This is notably different from other trialkylsilyl functionalized organic semiconductors, such as TIPS-pentacene, which are amorphous upon spin-coating. The spin-cast film accesses the known crystal structure with the (001) plane parallel to the substrate; however, the dropcast film adopts a slightly shifted structure from the solved bulk crystal structure (Fig. 5g), possibly explaining why the drop-cast devices show inferior transfer characteristics compared to spin-cast devices. Both spin- and drop-cast films are unaffected by post-deposition processing through either SVA or TA (Fig. 5b, c, e and f, respectively). The lack of polymorphic transformation as a result of post-deposition processing is supported by the presence of short interlayer contacts observed in the crystal structure, which have been shown to screen for kinetically stable polymorphs in organic molecular solids.^75^

## Conclusions

We presented a robust and simple strategy for inducing π-stacking in aromatic chromophores by using a universal aromatic core molecule containing a trialkylsilylethynyl group to tune crystal packing, to which one can attach a wide variety of π-conjugated pendants. Each pendant screened here yielded soluble, easily crystallized derivatives that adopted planar, π-stacked arrays in the solid state. Selecting the BDT pendant to demonstrate optimization of crystal packing for OFET applications, simple manipulation of the trialkylsilyl group afforded a champion material, **3b–h**, which exhibited hole mobility as high as 1.6 cm^2^ V^–1^ s^–1^. As π-stacking was observed in all derivatives, careful choice of pendant group may allow this strategy to be applied beyond OFET materials, as molecules with more localized orbitals, like those observed in **3d** and **3e**, could ostensibly be used for photonic applications^76^ by undertaking crystal engineering efforts to specifically optimize the crystal packing or aggregation for the desired application.

## Conflicts of interest

The authors have no conflicts to declare.

## Supplementary Material

Supplementary information

Crystal structure data
