# Supplementary material for: Computationally aided design of a high-performance organic semiconductor: the development of a universal crystal engineering core
Source: Chem Sci. 2019 Oct 7;10(45):10543–9. doi: 10.1039/c9sc02930c (PMC6988752; doi:10.1039/c9sc02930c)
Supplement: Supplementary file 1 [file SC-010-C9SC02930C-s001.pdf]

Electronic Supplementary Material (ESI) for Chemical Science.
This journal is © The Royal Society of Chemistry 2019

Computationally Aided Design of a High-Performance Organic Semiconductor via the Development of a Universal Crystal Engineering Core

Anthony J. Petty II,[a] Qianxiang Ai, [a] Jeni C. Sorli, [b] Hamna F. Haneef, [c] Geoffrey E. Purdum,[b] Alex Boehm, [a] Devin B. Granger, [a] Kaichen Gu, [b] Carla Patricia Lacerda Rubinger, [d] Sean R. Parkin, [a] Kenneth R. Graham, [a] Oana D. Jurchescu, [c] Yueh-Lin Loo, [ae] Chad Risko, [af] John E. Anthony *[af]

a. Department of Chemistry, University of Kentucky, Lexington, Kentucky, 40506-0055, USA
b. Department of Chemical and Biological Engineering, Princeton University, Princeton, New Jersey, 08544, United States
c. Department of Physics and Center for Functional Materials, Wake Forest University, USA
d. Physics and Chemistry Institute, Federal University of Itajubá, 37500-903, Itajubá, MG, Brazil.
e. Andlinger Center for Energy and the Environment, Princeton University, Princeton, New Jersey 08544, USA
f. Center for Applied Energy Research, University of Kentucky, Lexington, Kentucky 40511, USA

Table of Contents

I. Synthesis **3**

II. Solid State and Optical Materials Characterization **10**

Figure S1 11
Figure S2 12
Figure S3 12

III. OFET Fabrication and Results **13**

Substrate Preparation – BGBC drop-cast devices 13

Device Fabrication – BGBC drop-cast devices 13

Device Fabrication – BGTC drop-cast devices 13

Aligned Drop-casting Method 13
Figure S4 14

Device Performance –Drop-cast devices 14
Figure S5 14
Figure S6 14

Device Fabrication – Spun-cast Devices 14

Device Performance – 3b-h spun-cast devices 15

IV. Computational Methods and Results **15**

Computation Details 15

Transition of Stereoisomers 16
Figure S9 16

Band Structures 17
Figure S10: 17
Figure S11: 18

V. Thin-Film Characterization **18**

GIXD Characterization 18

Photoelectron Spectroscopies 18
Figure S12 18
Figure S13 19

Thin Film Fabrication for PES 19

PES Characterization 19

References **19**

I. Synthesis

All solvents were purchased in bulk from VWR. Anhydrous THF was purchased from Sigma Aldrich. All other chemicals were purchased from commercial sources and used as received unless otherwise noted. 2,6-diiodo benzodithiophene quinone[1], benzodithiophene[2], iodoazulene[3], tetrathio-fulvalene[4], tri(methyl)stannyl tetrathiofulvalene[5], and (*n*-octyl)bis(*iso*propyl)silyl acetylene[6] were synthesized according to literature procedures. LiMg(*n*-butyl)$_2$(*iso*propyl) was prepared according to the method of Struc and Sosnicki.[7] NMR spectra were measured on a 400 MHz Varian Unity spectrometer. Chemical shifts of each spectrum are reported in ppm and referenced to their corresponding deuterated solvents as listed. GC-MS was measured using a Bruker Scion-SQ GC-MS with an EI source. X-ray diffraction data were collected at low temperature on either Bruker-Nonius X8 Proteum or Bruker D8 Venture kappa-axis diffractometers using CuK(alpha) or MoK(alpha) X-rays. Raw data were integrated, scaled, merged and corrected for Lorentz-polarization effects using the APEX2 (X8)[8] or APEX3 (D8)[9] programs Corrections for absorption were applied using SADABS.[10] Structures were solved by direct methods (SHELXT[11]) and refinements were carried out against F^2 by weighted full-matrix least-squares (SHELXL[12]). Hydrogen atoms were found in difference maps, but subsequently placed at idealized positions and refined using a riding model. Non-hydrogen atoms were refined with anisotropic displacement parameters. Atomic scattering factors were taken from the International Tables for Crystallography.[13]

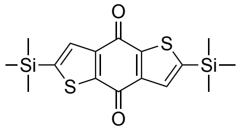

2,6-bis(trimethylsilyl) benzodithiophene quinone **(1)**: A solution of benzodithiophene quinone (2 g, 0.009 mol, 1 eq) in anhydrous THF (100 mL) was added trimethylsilyl chloride (4.4 g, 0.04 mol, 4.5 eq). The solution was cooled to 0° C and LiHMDS (1.0 M in THF, 20 mL, 0.02 mol, 2.2 eq) dropwise via an addition funnel. The reaction mixture was stirred for 1 hr, quenched with water and extracted with dichloromethane, dried with $MgSO_4$, and the solvent was removed on a rotovap. The crystalline yellow solid was redissolved in dichloromethane and passed through a short plug of silica eluting with dichloromethane. The solvent was removed on a rotovap affording a bright yellow crystalline powder (2.4 g, 0.0066 mol, 73%).^{1}HNMR (400 MHz; $CDCl_3$): δ 7.73 (s, 2H), 0.38 (s, 18H).^{13}C NMR (101 MHz; $CDCl_3$): δ 174.9, 151.7, 149.2, 143.9, 132.9 -0.3. MS (EI): m/z Calcd for $C_{16}H_{20}O_2S_2Si_2$ $[M]^+$: 364.04. Anal. Calcd for $C_{16}H_{20}O_2S_2Si_2$: C, 52.71, H, 5.53; found: C, 52.44 H, 5.25.

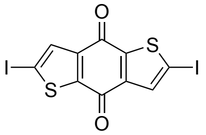

2,6-diiododbenzodithiophene quinone **(2)**: 1 (2 g, 0.0055 mol, 1 eq) was dissolved in dichloromethane (50 mL) and cooled to 0° C. ICl (1.0 M in DCM, 16 mL, 0.016 mol, 2.9 eq) was added dropwise and the reaction mixture was stirred overnight. The reaction mixture was then filtered, affording a highly crystalline orange solid, which was recrystallized from chloroform (2.22 g, 0.0047 mol, 85%) ^{1}HNMR (400 MHz; $CDCl_3$): δ 7.78 (s, 2H). , ^{13}C NMR (101 MHz; $CDCl_3$): δ 172.0, 143.3, 136.1, 86.2. Anal. Calcd (%) for $C_{10}H_2S_2I_2O_2$: C, 25.44%, H 0.43%; found: C, 25.33; H, 0.57.

General Procedure for the alkyne addition: In a flame dried round bottomed flask under nitrogen, the trialkylsilylacetylene (4 eq) was dissolved in 15 mL heptane and 6 mL anhydrous THF and cooled to 0° C in an ice bath. To this, *n*-butyl lithium (2.5 M in hexanes, 3.6 eq) was added dropwise and stirred for ~20 minutes, followed by the addition of 2 (1 eq), and was stirred overnight. The following morning the reaction was quenched with water and extracted into ether, dried with $MgSO_4$, filtered and the solvent was removed on a rotovap. The yellow oil was purified via a short plug of silica, eluting first with hexanes, then with 1:1 hexanes:DCM to recover the diol. The diol was then dissolved in ~1 mL acetone and 10 mL MeOH. $SnCl_2$•$2H_2O$ (2.8 g, 0.0125 eq, 5 eq) dissolved in ~5 mL 10% $HCl_{(aq)}$ was added dropwise to the dissolved diol, and the reaction was stirred for ~2 hours, during which the product oiled out of solution. Upon completion via TLC, water was added and the reaction was extracted with hexanes, dried with $MgSO_4$, filtered and the solvent was removed by rotary evaporation. Crude products were purified via column chromatography (silica gel, eluting with 100% hexanes).

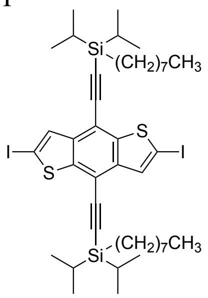

2,6-diiodo-4,8-bis(*n*-octyldi(isopropyl)ethynyl)-benzodithiophene **(2-no)**: *n*-octyldi(isopropyl)-acetylene (2.6 g, 0.0103 mol, 4 eq) and 2 (1.2 g, 0.0025 mol, 1 eq), and 2 were reacted according to the standard procedure, giving the final product as a pale yellow oil. (1.7 g, 0.0018 mol, 71%). ^1H NMR (400 MHz; C_6D_6): δ 7.86 (s, 2H), 1.68-1.62 (m, 4H), 1.47-1.31 (m, 23H), 1.25-1.16 (m, 27H), 1.08 (dt, J = 8.4, 6.6 Hz, 5H), 0.92 (q, J = 4.4 Hz, 7H), 0.81-0.77 (m, 4H). ^{13}C NMR (101 MHz; C_6D_6): δ 145.6, 139.5, 133.3, 109.8, 103.3, 102.3, 81.9, 34.3, 32.5, 29.89, 29.86, 25.2, 23.2, 18.7, 18.5, 14.5, 12.2, 10.4, 103.3, 102.3, 81.9, 34.3, 32.5, 29.89, 29.86, 25.2, 23.2, 18.7, 18.5, 14.5, 12.2, 10.4. HRMS (Direct-injection EI, 70 eV) m/z Calcd for $C_{42}H_{64}I_2S_2Si_2$: 942.2077; found 942.2100

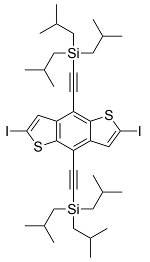

2,6-diiodo-4,8-bis(tri(iso-butyl)silylethynyl)-benzodithiophene **(2-i):** *tri(*isobutyl)silyl-acetylene (1.1 g, 0.0022 mol, 4 eq), *n*-butyl lithium (2.5 M in hexanes, 0.8 mL, 0.002 mol 3.6 eq) and **2** (0.26 g, 0.00055 mol, 1 eq), were reacted according to the standard procedure, giving the final product as a crystalline white solid (0.4 g, 81%). ^1H NMR (400 MHz; C_6D_6): δ 7.89 (s, 2H), 2.12-1.99 (m, 6H), 1.13 (d, *J* = 6.6 Hz, 36H), 0.80 (d, *J* = 6.9 Hz, 12H). ^{13}C NMR (101 MHz; C_6D_6): δ 145.4, 139.8, 133.5, 109.9, 106.0, 102.4, 81.8, 26.6, 25.6, 25.4. HRMS (Direct-injection EI, 70 eV) m/z Calcd for $C_{38}H_{56}I_2S_2Si_2$: 886.1451; found 886.1430

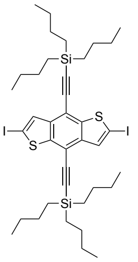

2,6-diiodo-4,8-bis(tri(*n*-butyl)silylethynyl)-benzodithiophene **(2-b):** tri*(*n-butyl)silyl-acetylene (2.1 g, 0.009 mol, 4 eq), *n*-butyl lithium (2.5 M in hexanes, 3.3 mL, 0.0084 mol 3.6 eq) and **2** (1 g, 0.002 mol, 1 eq), were reacted according to the standard procedure, giving the final product as a crystalline white solid (1.4 g, 75%). ^{1}H NMR (400 MHz; C_6D_6): δ 7.84 (s, 2H), 1.63-1.56 (m, 12H), 1.46 (dq, J = 14.8, 7.3 Hz, 12H), 1.00 (t, J = 7.3 Hz, 18H), 0.80 (m, 12H). ^{13}C NMR (101 MHz; C_6D_6): δ 145.5, 139.5, 133.4, 109.9, 104.8, 101.9, 81.8, 26.92, 26.88, 14.2, 13.5. HRMS (Direct-injection EI, 70 eV) m/z Calcd for $C_{38}H_{56}I_2S_2Si_2$: 886.1451; found 886.1440

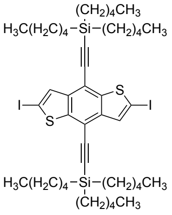

2,6-diiodo-4,8-bis(tri(*n*-pentyl)silylethynyl)-benzodithiophene **(2-p):** tri*(*n-pentyl)silyl-acetylene (0.42 g, 0.0016 mol, 4 eq), *n*-butyl lithium (2.5 M in hexanes, 0.6 mL, 0.0015 mol 3.6 eq) and **2** (0.2 g, 0.0004 mol, 1 eq), were reacted according to the standard procedure, giving the final product as a pale yellow oil (0.24 g, 64%). δ ^{1}H NMR (400 MHz; C_6D_6): δ 7.84 (d, J = 0.3 Hz, 2H), 1.68-1.60 (m, 12H), 1.47-1.38 (m, 25H), 0.98 (t, J = 7.2 Hz, 18H), 0.83-0.79 (m, 12H). ^{13}C NMR (101 MHz; C_6D_6): δ ^{13}C NMR (101 MHz; C_6H_6) *δ* 145.5, 139.5, 133.3, 109.9, 104.9, 101.9, 81.8, 36.1, 24.3, 22.9, 14.4, 13.7. HRMS (Direct-injection EI, 70 eV) m/z Calcd for $C_{44}H_{68}I_2S_2Si_2$: 970.2390; found 970.2400.

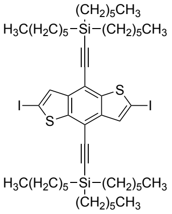

2,6-diiodo-4,8-bis(tri(*n*-hexyl)silylethynyl)-benzodithiophene **(2-h):** tri*(*n-hexyl)silyl-acetylene (2.5 g, 0.008 mol, 4 eq), *n*-butyl lithium (2.5 M in hexanes, 3.3 mL, 0.0084 mol 3.6 eq) and **2** (1 g, 0.002 mol, 1 eq), were reacted according to the standard procedure, giving the final product as a pale yellow oil (1.4 g, 68%). ^{1}H NMR (400 MHz; $CDCl_3$): δ 7.73 (s, 2H), 1.54-1.48 (m, 13H), 1.45-1.32 (m, 35H), 0.91 (t, J = 6.9 Hz, 18H), 0.76 (dd, J = 9.3, 7.0 Hz, 12H). ^{13}C NMR (101 MHz; $CDCl_3$): δ 145.1, 139.0, 133.1, 109.4, 105.0, 101.1, 81.1, 77.5, 77.2, 76.8, 33.3, 31.8, 24.2, 22.8, 14.4, 13.5. HRMS (Direct-injection EI, 70 eV) m/z Calcd for $C_{50}H_{80}I_2S_2Si_2$: 1054.3329; found 1054.3301.

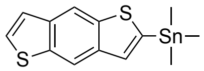

2-tri(methyl)stannyl benzodithiophene: A flame dried round bottomed flask was charged with benzodithiophene (0.5 g, 0.0026 mol, 1 eq) and anhydrous THF (50 mL) and was cooled in an acetone/dry ice bath for ~20 minutes. To this, *n*-butyl lithium (2.5 M in hexanes, 1.15 mL, 0.0028 mol, 1.1 eq) was added dropwise and stirred for ~60 minutes, followed by the addition of tri(methyl)stannyl chloride (1 M in hexane, 3.4 mL, 0.0034 mol, 1.3 eq). The reaction was stirred for 10 minutes, then removed from the bath and allowed to warm to room temperature. The reaction was quenched with water, extracted with hexanes, the organic layer was dried with $MgSO_4$, filtered and the solvent was removed leaving behind a silvery solid which was used as prepared (0.75 g, 0.0021 mol, 82%).

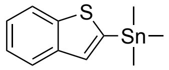

2-tri(methyl)stannyl benzothiophene: A flame dried round bottomed flask was charged with benzothiophene (0.5 g, 0.0037 mol, 1 eq) and anhydrous THF (50 mL) and was cooled in an acetone/dry ice bath for ~20 minutes. To this, *n*-butyl lithium (2.5 M in hexanes, 1.6 mL, 0.0041 mol, 1.1 eq) was added dropwise and stirred for ~60 minutes, followed by the addition of tri(methyl)stannyl chloride (1 M in hexane, 4.8 mL, 0.0048 mol, 1.3 eq). The reaction was stirred for 10 minutes, then removed from the bath and allowed to warm to room temperature. The reaction was quenched with water, extracted with hexanes, the organic layer was dried with $MgSO_4$, filtered and the solvent was removed leaving behind a pale yellow oil which was used without further purification (0.98 g, 0.0033 mol, 90%).

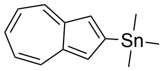

2-tri(methyl)stannyl azulene: Using a modification of the procedure by Ito *et al*[14], A flame dried round bottomed flask was charged with iodoazulene (0.12 g, 0.00047 mol, 1 eq) and anhydrous THF (10 mL) and was cooled in an acetone/dry ice bath for ~20 minutes. A solution of LiMg(*n*-butyl)$_2$(*isop*ropyl) (0.15 M in THF, 3.5 mL, 0.00051 mol, 1.1 eq) was added dropwise and stirred for 20 minutes, then trimethylstannyl chloride (1 M in hexane, 1.5 mL, 0.0015 mol, 3.2 eq). The reaction was stirred for 10 minutes, then removed from the bath and allowed to warm to room temperature. The reaction was quenched with water, extracted with hexanes, the organic layer was dried with $MgSO_4$, filtered and the solvent was removed leaving behind a blue oil which slowly crystallized, which was used without further purification (0.09 g, 0.00031 mol, 66%).

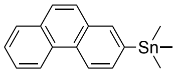

2-tri(methyl)stannyl phenantherene: A flame dried round bottomed flask was charged with 2-bromophenanthene (0.4 g, 0.0015 mol, 1 eq) and anhydrous THF (40 mL) and was cooled in an acetone/dry ice bath for ~20 minutes. To this, *n*-butyl lithium (2.5 M in hexanes, 0.68 mL, 0.0017 mol, 1.1 eq) was added dropwise and stirred for ~60 minutes, followed by the addition of tri(methyl)stannyl chloride (1 M in hexane, 2.0 mL, 0.0020 mol, 1.3 eq). The reaction was stirred for 10 minutes, then removed from the bath and allowed to warm to room temperature. The reaction was quenched with water, extracted with hexanes, the organic layer was dried with $MgSO_4$, filtered and the solvent was removed leaving behind an off white solid which was used without further purification (0.45 g, 0.0013 mol, 84%)

Standard procedure for Stille Coupling: A flame dried sealed tube was charged with 3 (1 eq) dissolved in toluene which was purged with N_2 for ~10 minutes. Then the stannane (2.5 eq), Pd_2dba_3 (0.05 eq), $AsPh_3$ (0.2 eq) was added, the tube was sealed, and was heated to 90° C overnight. The reaction mixture was cooled to room temperature, diluted with toluene, and filtered through celite. The solvent was removed and the remaining solid was triturated with hexanes, filtered, and the solid was then purified via column chromatography. The pure fractions were combined, the solvent was removed, and the solid was recrystallized.

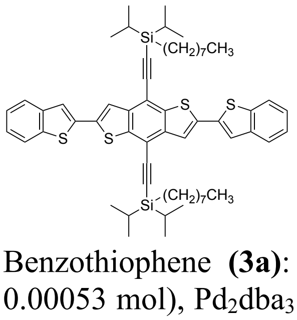

Benzothiophene **(3a)**: **2-no** (0.2 g, 0.00021 mol), trimethylstannyl benzothiophene (0.16 g, 0.00053 mol), Pd_2dba_3 (0.01 g, 1.1 x 10^{-5} mol), $AsPh_3$ (0.013 g, 4.4 x 10^{-5} mol) were reacted via the standard procedure. The crude product was purified by column chromatography (silica gel, 10:1 cyclohexane:toluene) and was then recrystallized from a mixture of hexanes and toluene to give the final product as gold needles (0.11 g, 0.00012 mol, 55%). ^{1}H NMR (400 MHz; $CDCl_3$): δ 7.84-7.79 (m, 4H), 7.74 (s, 2H), 7.57 (s, 2H), 7.40-7.33 (m, 4H), 1.68-1.60 (m, 4H), 1.52-1.21 (m, 49H), 0.86-0.81 (m, 10H). ^{13}C NMR (101 MHz; $CDCl_3$): δ 140.4, 140.1, 139.7, 139.3, 138.9, 136.9, 125.1, 124.8, 123.8, 122.16, 122.09, 120.5, 111.6, 102.9, 101.9, 33.9, 32.0, 29.42, 29.38, 24.7, 22.7, 18.5, 18.2, 14.1, 11.8, 10.1 Anal. Calcd (%) for $C_{58}H_{74}S_4Si_2$: C, 72.90; H 7.81; found: C, 72.66; H, 7.71. Submitted to the Cambridge Crystallographic Data Centre as CCDC 1833615

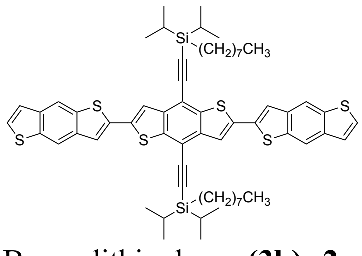

Benzodithiophene **(3b)**: **2-no** (0.2 g, 0.00021 mol), trimethylstannyl benzodithiophene (0.19 g, 0.00053 mol), Pd_2dba_3 (0.01 g, 1.1 x 10^{-5} mol), $AsPh_3$ (0.013 g, 4.4 x 10^{-5} mol) were reacted via the standard procedure. The crude product was purified by column chromatography (silica gel, 10:1 cyclohexane:toluene) and was then recrystallized from a mixture of hexanes and toluene to give the final product as gold needles (0.15 g, 0.00014 mol, 68%). ^{1}H NMR (400 MHz; CD_2Cl_2/CS_2): δ 8.27 (s, 2H), 8.24 (s, 2H), 7.73 (s, 2H), 7.61 (s, 2H), 7.51 (d, J = 5.5 Hz, 2H), 7.39-7.37 (m, 2H), 1.72-1.64 (m, 4H), 1.54-1.24 (m, 53H), 0.90-0.85 (m, 10H). ^{13}C NMR (101 MHz; CD_2Cl_2/CS_2): δ 141.1, 139.8, 139.6, 138.7, 138.33, 138.29, 137.8, 137.5, 128.3, 123.5, 121.9, 121.3, 117.7, 117.0, 112.2, 103.7, 102.5, 34.7, 32.8, 30.2, 25.4, 23.6, 19.0, 18.8, 14.7, 12.6, 10.8 Anal. Calcd (%) for $C_{62}H_{74}S_6Si_2$: C, 69.74; H 7.99; found: C, 69.74; H, 7.66. Submitted to the Cambridge Crystallographic Data Centre as CCDC 1833616

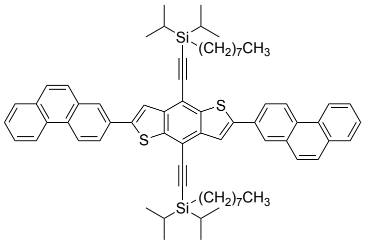

Phenantherene **(3d)**: **2-no** (0.2 g, 0.00021 mol), trimethylstannyl phenatherene (0.18 g, 0.00053 mol), Pd_2dba_3 (0.01 g, 1.1 x 10^{-5} mol), $AsPh_3$ (0.013 g, 4.4 x 10^{-5} mol) were reacted via the standard procedure. The crude product was purified by column chromatography (silica gel, 10:1 cyclohexane:toluene) and was then recrystallized from a mixture of hexanes and toluene to give the final product as a bright yellow crystalline solid (0.14 g, 0.00013 mol, 64%). ^{1}H NMR (400 MHz; $CDCl_3$): δ 8.75 (d, J = 8.8 Hz, 2H), 8.69 (d, J = 8.2 Hz, 2H), 8.21 (d, J = 1.8 Hz, 2H), 8.04 (dd, J = 8.6, 2.0 Hz, 2H), 7.94 (d, J = 1.2 Hz, 2H), 7.90 (d, J = 7.9 Hz, 2H), 7.81 (d, J = 3.6 Hz, 4H), 7.70-7.66 (m, 2H), 7.60 (td, J = 7.4, 0.9 Hz, 2H), 1.72-1.66 (m, 4H), 1.53-1.21 (m, 50H), 0.91-0.87 (m, 4H), 0.82-0.79 (m, 6H)^{13}C NMR (101 MHz; $CDCl_3$): δ 153.7, 151.2, 149.3, 145.4, 143.6, 140.5, 139.6, 132.29, 132.26, 132.23, 132.13, 130.3, 130.0, 128.7, 127.78, 127.76, 126.94, 126.89, 126.85, 126.81, 126.18, 126.15, 124.6, 123.5, 122.75, 122.71, 119.0, 111.6, 102.38, 102.34, 34.0, 32.0, 29.46, 29.43, 29.40, 24.8, 22.7, 18.6, 18.3, 14.0, 11.88, 11.87, 10.2 Anal. Calcd (%) for $C_{70}H_{82}S_2Si_2$: C, 80.56; H 7.92; found: C, 80.96; H, 7.89. Submitted to the Cambridge Crystallographic Data Centre as CCDC 1833617.

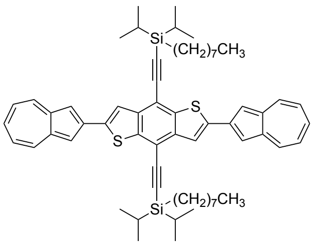

Azulene **(3e)**: **2-no** (0.09 g, 9.5 x 10^{-5} mol), trimethylstannyl azulene (0.07 g, , 24 x 10^{-5} mol), Pd_2dba_3 (0.005 g 0.5 x 10^{-5} mol), $AsPh_3$ (0.007 g, 2.2 x 10^{-5} mol) were reacted via the standard procedure. However, this molecule was not triturated with hexane. The crude product was purified by column chromatography (silica gel, 10:1 hexane:dichloromethane) and was then recrystallized from a mixture of hexanes and toluene to give the final product as iridescent green needles (0.038 g, , 3.9 x 10^{-5} mol, 41%). ^{1}HNMR (400 MHz; $CDCl_3$): δ 8.29 (d, J = 9.2 Hz, 4H), 8.04 (s, 2H), 7.66 (s, 4H), 7.52 (t, J = 9.9 Hz, 2H), 7.18 (t, J = 9.9 Hz, 4H), 1.70-1.64 (m, 4H), 1.52-1.19 (m, 50H), 0.89-0.79 (m, 10H).^{13}C NMR (101 MHz; $CDCl_3$): δ 142.9, 142.4, 141.62, 141.43, 140.0, 137.2, 136.6, 124.5, 121.3, 115.0, 111.9, 102.59, 102.58, 77.5, 77.2, 76.8, 34.2, 32.2, 29.65, 29.61, 24.9, 22.9, 18.7, 18.5, 14.3, 12.1, 10.4 Anal. Calcd (%) for $C_{62}H_{78}S_2Si_2$: C, 78.92; H 8.33; found: C, 79.88; H, 8.21. Submitted to the Cambridge Crystallographic Data Centre as CCDC 1833619.

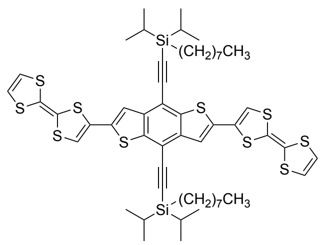

Tetrathiofulvalene **(3c)**: **2-no** (0.2 g, 0.00021 mol), trimethylstannyl tetrathiofulvalene (0.19 g, 0.00053 mol), Pd_2dba_3 (0.01 g, 1.1 x 10^{-5} mol), $AsPh_3$ (0.013 g, 4.4 x 10^{-5} mol) were reacted via the standard procedure. The crude product was purified by column chromatography (silica gel, 4:1 hexanes:DCM) and was then recrystallized from a mixture of hexanes and toluene to give the final product as a dark needles (0.12 g, 0.00011 mol, 52%). ^{1}H NMR (400 MHz; D_6-acetone/CS_2): δ 7.29 (s, 2H), 6.77 (s, 2H), 6.47 (s, 4H), 1.64-1.57 (m, 4H), 1.51-1.44 (m, 4H), 1.40-1.19 (m, 49H), 0.91-0.88 (m, 7H), 0.85-0.81 (m, 4H).^{13}C NMR (101 MHz; D_6-acetone/CS_2): δ 140.5, 139.7, 137.2, 129.7, 122.0, 120.08, 120.07, 119.4, 115.1, 112.3, 107.7, 104.0, 102.4, 34.8, 32.9, 29.8, 25.5, 23.8, 19.2, 15.0, 12.8, 10.9Anal. Calcd (%) for $C_{54}H_{70}S_{10}Si_2$: C, 59.18; H 6.44; found: C, 59.42; H, 6.36. Submitted to the Cambridge Crystallographic Data Centre as CCDC 1833618.

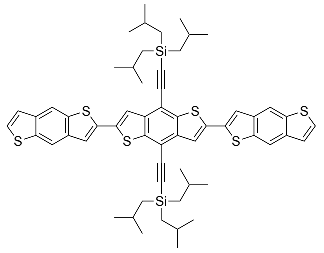

Tri(*iso*butyl)silylethynyl benzodithiophene trimer (**3b-i**): **2-i** (0.1 g, 0.00011 mol), trimethylstannyl benzodithiophene (0.09 g, 0.00026 mol), Pd_2dba_3 (0.01 g, 1.1 x 10^{-5} mol), $AsPh_3$ (0.013 g, 4.4 x 10^{-5} mol) were reacted via the standard procedure. The crude product was purified by column chromatography (silica gel, DCM) and was then recrystallized from toluene to give the final product as yellow/orange needles (0.06 g, 55%). ^{1}H NMR (400 MHz; CD_2Cl_2/CS_2): δ 8.30-8.29 (m, 2H), 8.30-8.27 (m, 2H), 7.77-7.73 (m, 2H), 7.65-7.61 (m, 2H), 7.56 (t, J = 6.9 Hz, 2H), 7.42 (d, J = 5.5 Hz, 2H), 2.25-2.13 (m, 6H), 1.43-1.15 (m, 38H), 1.13-0.91 (m, 13H).^{13}C NMR (101 MHz; CD_2Cl_2/CS_2): δ 141.1, 139.9, 139.7, 138.7, 138.43, 138.39, 138.0, 137.6, 128.4, 123.6, 121.9, 121.3, 117.8, 117.1, 112.3, 106.1, 102.7, 27.2, 26.1, 25.8. Anal. Calcd (%) for $C_{59}H_{66}S_6Si_2$: C, 69.09; H, 6.68; found: C, 68.71; H, 6.75. Submitted to the Cambridge Crystallographic Data Centre as CCDC 1922872.

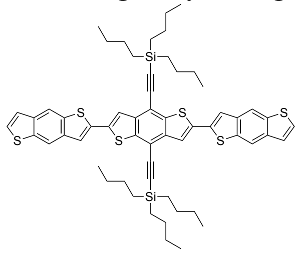

Tri(*n*-butyl)silylethynyl benzodithiophene trimer (**3b-b**): **2-b** (0.3 g, 0.0003 mol), trimethylstannyl benzodithiophene (0.27 g, 0.00078 mol), Pd_2dba_3 (0.03 g, 3.3 x 10^{-5} mol), $AsPh_3$ (0.04 g, 13 x 10^{-5} mol) were reacted via the standard procedure. The crude product was

purified by column chromatography (silica gel, DCM) and was then recrystallized from a mixture of hexane and toluene to give the final product as yellow/orange needles (0.19 g, 64%). ^{1}H NMR (400 MHz; CD_2Cl_2/CS_2): δ 8.26 (s, 2H), 8.23 (s, 2H), 7.71 (s, 2H), 7.60 (s, 2H), 7.51 (d, J = 5.5 Hz, 2H), 7.38 (dd, J = 5.6, 0.7 Hz, 2H), 1.68-1.51 (m, 25H), 1.07 (t, J = 7.2 Hz, 18H), 0.92-0.88 (m, 12H). ^{13}C NMR (101 MHz; CD_2Cl_2/CS_2): δ 141.2, 139.70, 139.65, 138.7, 138.39, 138.36, 137.9, 137.6, 128.4, 123.6, 121.9, 121.3, 117.8, 117.1, 112.3, 105.3, 102.0, 100.4, 27.5, 27.2, 14.7, 13.9. Anal. Calcd (%) for $C_{58}H_{66}S_6Si_2$: C, 68.86; H, 6.58; found: C, 68.99; H, 6.76. Submitted to the Cambridge Crystallographic Data Centre as CCDC 1922873.

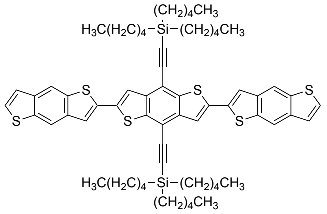

Tri(n-pentyl)silylethynyl benzodithiophene trimer (**3b-p**): **2-p** (0.15 g, 0.00015 mol), trimethylstannyl benzodithiophene (0.13 g, 0.000375 mol), Pd_2dba_3 (0.007 g, 0.75 x 10^{-5} mol), $AsPh_3$ (0.001 g, 3 x 10^{-5} mol) were reacted via the standard procedure. The crude product was purified by column chromatography (silica gel, 10:1 Hexane:DCM) and was then recrystallized from a mixture of hexane and toluene to give the final product as long yellow needles (0.11 g, 70%) ^{1}H NMR (400 MHz; CD_2Cl_2/CS_2): δ 8.25 (s, 2H), 8.22 (s, 2H), 7.70 (s, 2H), 7.59 (s, 2H), 7.51 (d, J = 5.5 Hz, 2H), 7.38 (d, J = 5.5 Hz, 2H), 1.68-1.61 (m, 12H), 1.55-1.43 (m, 24H), 1.02 (t, J = 7.0 Hz, 18H), 0.90-0.86 (m, 13H). ^{13}C NMR (101 MHz; CD_2Cl_2/CS_2): δ 141.3, 139.7, 138.7, 138.4, 137.9, 137.6, 128.4, 123.6, 121.9, 121.3, 117.8, 117.1, 112.3, 105.3, 102.1, 100.3, 36.6, 24.7, 23.5, 15.0, 14.2. Anal. Calcd (%) for $C_{64}H_{78}S_6Si_2$: C, 70.15; H, 7.17; found: C, 70.22; H, 7.12. Submitted to the Cambridge Crystallographic Data Centre as CCDC 1922871.

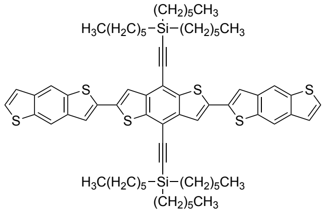

Tri(n-hexyl)silylethynyl benzodithiophene trimer (**3b-h**): **2-h** (0.4 g, 0.00038 mol), trimethylstannyl benzodithiophene (0.33 g, 0.001 mol), Pd_2dba_3 (0.017 g, 1.9 x 10^{-5} mol), $AsPh_3$ (0.023 g, 7.5 x 10^{-5} mol) were reacted via the standard procedure. The crude product was purified by column chromatography (silica gel, 10:1 hexane:DCM) and was then recrystallized from a mixture of hexane and toluene to give the final product as orange plates (0.28 g, 62%) ^{1}H NMR (400 MHz; CD_2Cl_2): δ 8.28 (s, 2H), 8.25 (s, 2H), 7.73 (s, 2H), 7.62 (s, 2H), 7.52 (d, J = 5.5 Hz, 2H), 7.38 (dd, J = 5.5, 0.5 Hz, 2H), 1.67-1.59 (m, 12H), 1.53-1.46 (m, 14H), 1.45-1.35 (m, 25H), 0.93-0.85 (m, 30H). ^{13}C NMR (101 MHz; CD_2Cl_2): δ 141.0, 139.60, 139.47, 138.7, 138.30, 138.22, 137.6, 137.4, 128.2, 123.4, 121.7, 121.0, 117.6, 116.9, 112.0, 105.4, 101.6, 54.3, 54.1, 53.8, 53.5, 53.3, 33.7, 32.2, 24.6, 23.1, 14.4, 13.8 Anal. Calcd (%) for $C_{70}H_{90}S_6Si_2$: C, 71.25; H, 7.69; found: C, 71.56; H, 7.61

II. Solid State and Optical Materials Characterization

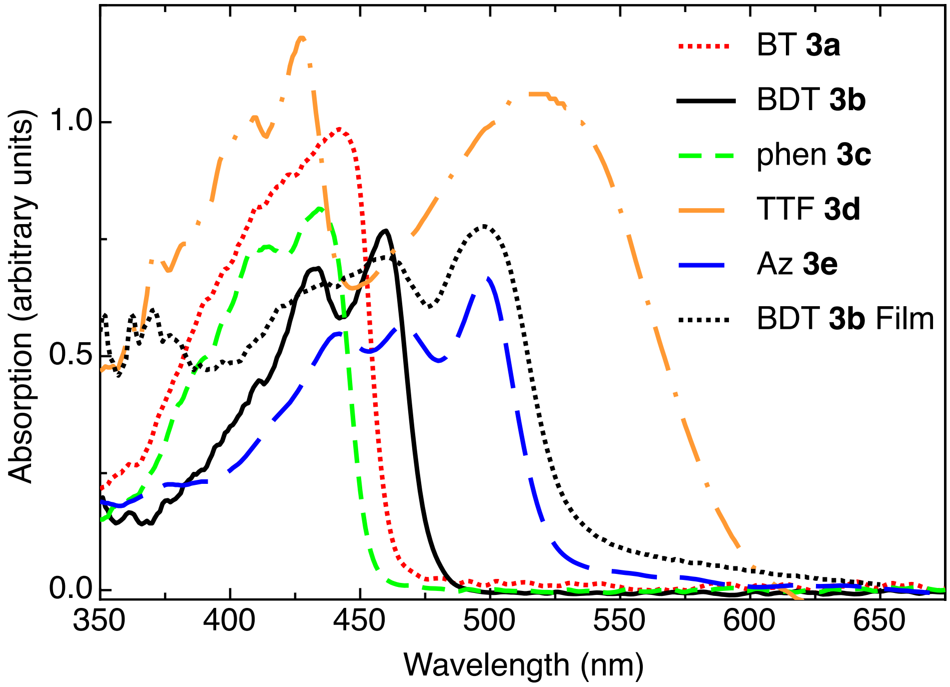

Figure S1: UV/Vis absorption spectra of compounds **3a-e** in toluene, along with the thin film absorption spectrum of **3b**.

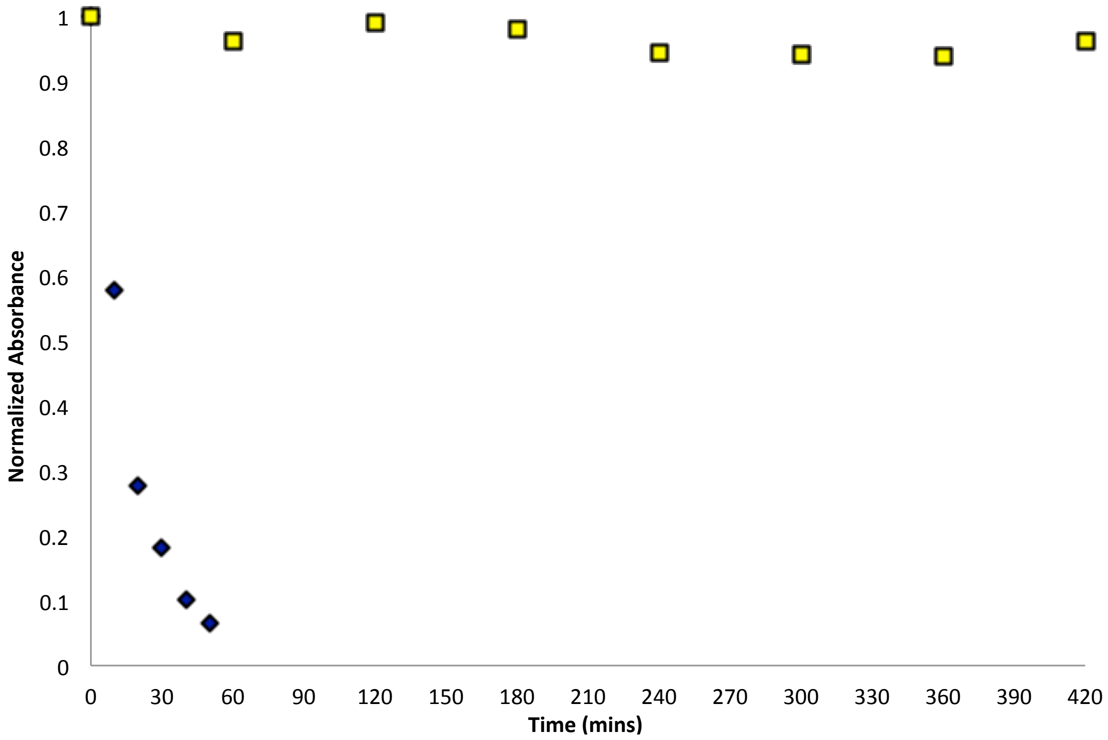

Figure S2: Relative photostability of **3b** (yellow) and TIPS Pentacene (blue) in toluene, as tracked by the absorbance at 459 nm for **3b** and TIPS Pentacene at 644 nm.

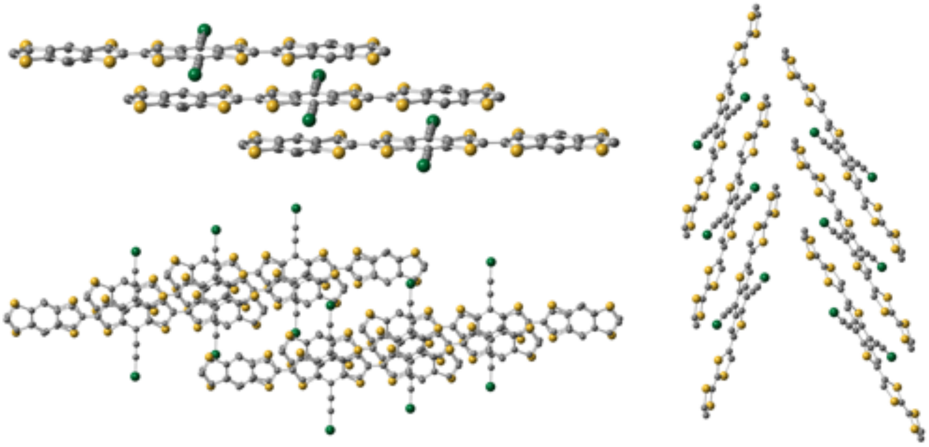

Figure S3: General crystal packing for derivatives **3(a-e)**, alkyl substituents removed for clarity. All derivatives adopted strongly π-stacked 1-D arrangements (top left) with derivatives **3(a-c, e)** showing significant close contacts between adjacent stacks (bottom left). The one-dimensional stacks of derivative **3d** packed in a nose-to-nose "chevron" fashion, as shown above right.

III. OFET Fabrication and Results

Substrate Preparation – BGBC drop-cast devices

Si wafers (Si(100)) with thermally grown SiO_2 (300 nm) were purchased from Process Specialties Inc., and used as the gate and gate dielectric, respectively, for thin-film transistors and for GIXD studies. The substrates were sonicated in deionized water, acetone, and isopropyl alcohol and then dried with nitrogen prior to transistor fabrication. For GIXD studies, films were prepared on cleaned, untreated Si/SiO_2 substrates. For 3b-b devices, the substrates were treated with hexamethyldisilazane (HMDS) by spin-coating at 2000 rpm for 20 seconds and then immediately placing them on a hot plate at 100°C for 1 minute. For 3b-h devices, which were made in the bottom-contact configuration, bottom contacts were prepared by depositing 2 nm of titanium, followed by 30 nm of gold through pre-defined stencil masks. These patterned substrates were immediately placed in a 7.5 mM PFBT/ethanol solution for 20 minutes upon removal from vacuum, then sonicated in ethanol and dried with nitrogen before deposition of the organic semiconductors.

Device Fabrication – BGBC drop-cast devices

Transistors were fabricated in a bottom-contact (PFBT treated Au), bottom-gate (highly doped Si) configuration with a 300-nm thick SiO_2 dielectric layer. Shadow masks were used to produced channels with a width of 2000 μm and a length of 100 μm. To deposit contacts, 2 nm of titanium and then 35 nm of gold were evaporated via e-beam under high vacuum at 1 $Ås^{-1}$ for both materials. These patterned substrates were immediately placed in a 7.5 mM PFBT/ethanol solution for 20 minutes upon removal from vacuum, then sonicated in ethanol and dried with nitrogen before deposition of the organic semiconductors, either using the modified drop-casting method described in the section below. All transistors were tested in air using an Agilent 4155C semiconductor parameter analyzer. Hole mobilities were estimated in the saturation regime at V_{DS} = -80 V. Threshold voltages were calculated by extrapolating the zero point of the square root of current versus the gate voltage in the saturation regime. On/off current ratios were calculated by taking the ratio of I_{SD} at -80 V (on) and at the turn-on voltage (off) from the transfer characteristics.

Device Fabrication – BGTC drop-cast devices

Top-contact (Au), bottom-gate (highly doped Si) thin-film transistors with a 300-nm SiO_2 dielectric layer were fabricated using substrates and films described above. Top contacts were deposited by thermally evaporating gold at high vacuum through a shadow mask at a rate of 1 $Ås^{-1}$ to a thickness of 50 nm atop thin films of **3b-b** (drop-casting method as described below). TEM grids (Ted Pella, Inc.) were used as shadow masks to define the channel dimensions of the transistors, with a channel width of 204 μm and a channel length of 50 μm.

Aligned Drop-casting Method

Films of **3b-h** and **3b-b** were deposited by a modified drop-casting method, similar to that reported by Soeda et al.[15] The substrate was placed on a hot plate at 45°C and a glass slide was placed atop the Si/SiO2 substrate with a 0.2 mm spacer on one end creating a wedge-shaped gap. For **3b-b** and **3b-h** films, 5 mg/mL and 10 mg/mL solutions in chloroform, respectively, were also heated to 45°C before being injected into the gap and allowed to dry. A scheme of the set-up is shown in Figure S2. After deposition, films of **3b-b** were subjected to thermal annealing at 150°C for 30 minutes; films of **3b-h** were not thermally annealed.

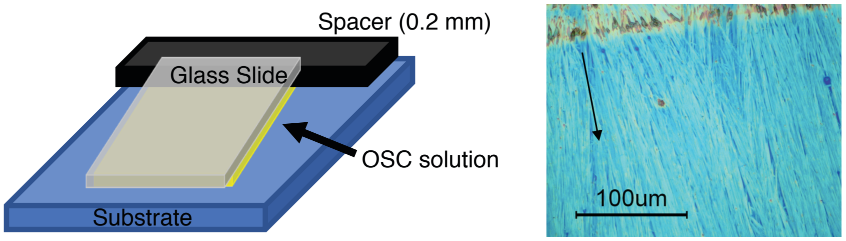

Figure S4: Modified dropcasting set-up to deposit thin films; optical image of a film prepared using this method with the direction of the evaporation front denoted by the black arrow

Device Performance –Drop-cast devices

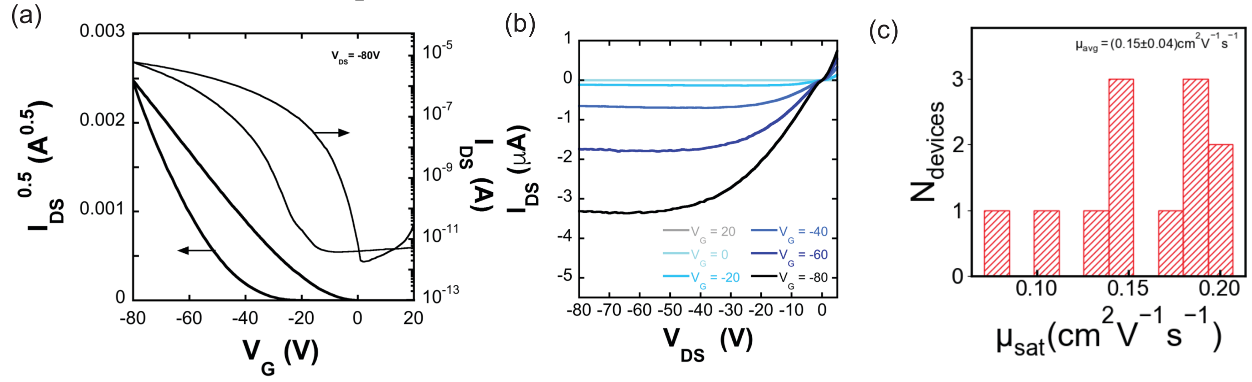

Figure S5: Representative transfer (a) and output (b) curves of drop cast devices of **3b-b** in the BGTC configuration. c) Histogram of saturation mobilities, inset shows average mobility.

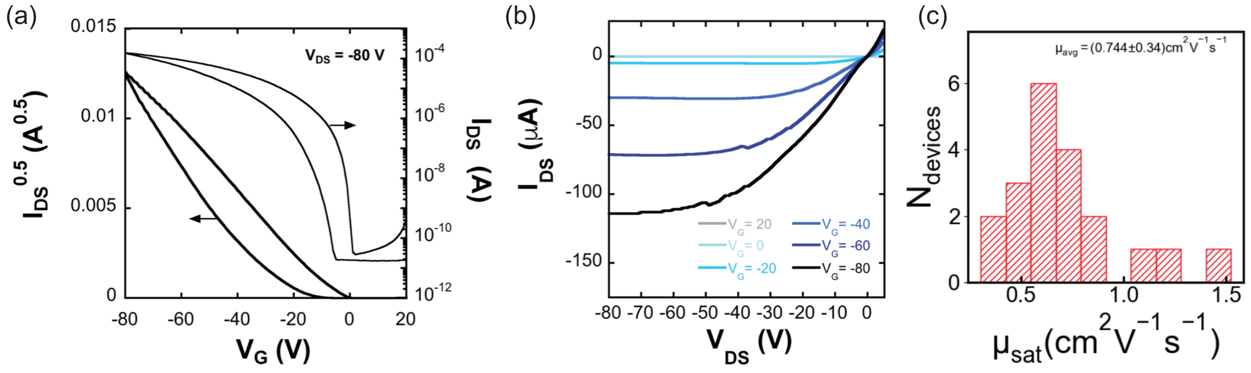

Figure S6: Representative transfer (a) and output (b) curves of drop cast devices of **3b-h** in the BGBC configuration c) Histogram of saturation mobilities, inset shows average mobility.

Device Fabrication – Spun-cast Devices

Two device architectures were adopted to perform FET measurements using compound **3b-h**; bottom-contact bottom-gate and bottom-contact top-gate. For the bottom-gate devices, a heavily doped n-type silicon wafer with a 200 nm thermally grown SiO_2 layer served as the gate electrode and gate dielectric, respectively. A 3 nm Ti layer was e-beam evaporated followed by thermal evaporation of 40 nm of Au through a shadow mask to form the source and drain

electrodes. The substrates were cleaned by immersing them in a warm acetone bath for 10 minutes followed by a warm isopropanol bath for 10 minutes. They were then exposed to UV ozone for 10 minutes followed by a DI water rinse. The semiconductor was then spun-cast onto the substrate at 1000 rpm using a 1.5 wt % solution of **3b-h** in chlorobenzene.

To fabricate the top-gate devices, parylene dielectric was deposited over the semiconductor film using a home-made reactor.[16] The deposition was performed inside an evacuated quartz tube placed under three different temperature zones. The parylene dimer was first vaporized at the first temperature zone maintained at 120 °C and pyrolyzed at the second zone at 700 °C to break into its monomers and was then allowed to polymerize at room temperature over the semiconductor film. Finally, 40 nm of Au was thermally evaporated through a shadow mask to form the top-gate.

Device Performance – 3b-h spun-cast devices

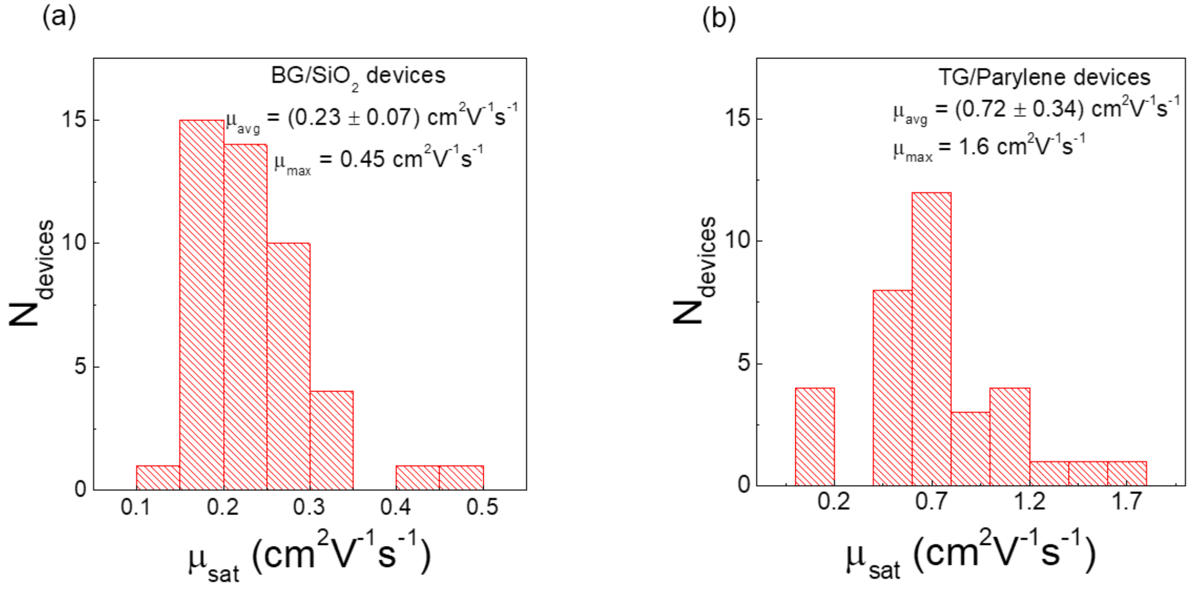

IV. Computational Methods and Results

Computation Details

For all systems with periodic boundary conditions, density functional theory (DFT) calculations were carried out with the Vienna Ab-initio Simulation Package (VASP),[17, 18] making use of the Perdew, Burke, and Ernzerhof (PBE)[19] exchange-correlation functional based on experimentally solved structures. The electron-ion interactions were described with the projector augment wave (PAW) method.[19, 20] The kinetic energy cutoff for the plane-wave basis set was set to 520 eV, and a Gaussian smearing with a width of 0.05 eV was employed. The convergence criterion of the total energy was set to 10^{-5} eV in the self-consistent loop. The Brillouin-zone was sampled with a 4×4×1 Γ-centered grid. High-symmetry points in the first Brillouin zone used for the band structure calculations were determined with the AFLOW software,[21] which follows the scheme proposed by Setyawan and Curtarolo.[22] Effective mass was calculated following the scheme implemented by Fonari.[23] For systems with open boundary conditions, the electronic structures were obtained via DFT calculations at wb97xd/def2svp level[24, 25] with Gaussian 16 package

(revision A.03).[26] Electronic couplings for dimer models, with geometries extracted from experimentally solved crystal structures, were calculated following the fragment orbital approach developed by Valeev and co-workers using the code from Ryno.[27, 28]

Transition of Stereoisomers

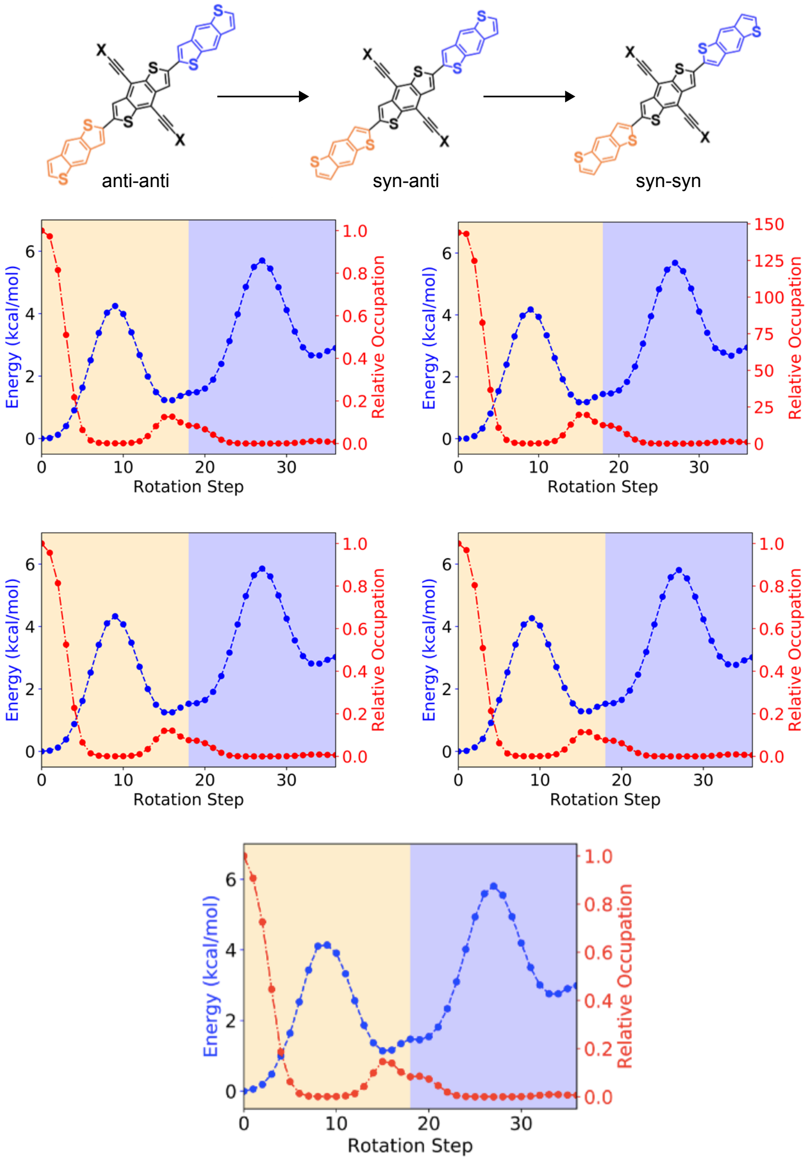

Figure S9: Transition between stereoisomers of (top-left) **3b**, (top-right) **3b-i**, (mid-left) **3b-b**, and (mid-right) **3b-p** and (bottom) **3b-h.** Potential surface scan for the transition between the stereoisomers of **3b-h** by sequentially rotating the dihedral angles between the BDT units as determined at the ωB97X-D/Def2SVP level of theory. The blue circles indicate the relative energy of a certain geometry with respect to the most stable geometry, and the relative occupation of that geometry is denoted by red circles.

For each molecule, the calculation is performed by fixing one of the two dihedrals between BDT units while optimizing the molecular geometry with the other dihedral is changed at 10 degrees per scan step. The results are similar for all derivatives in this study.

Band Structures

The electronic band structures for 3b derivatives (Figure S10) are calculated following the methods specified in Computational Details. The red dots indicate the sampling points along the path between high symmetry points in the first Brillouin-zone, and the five bands above/below the Fermi level are fitted with blue splines.

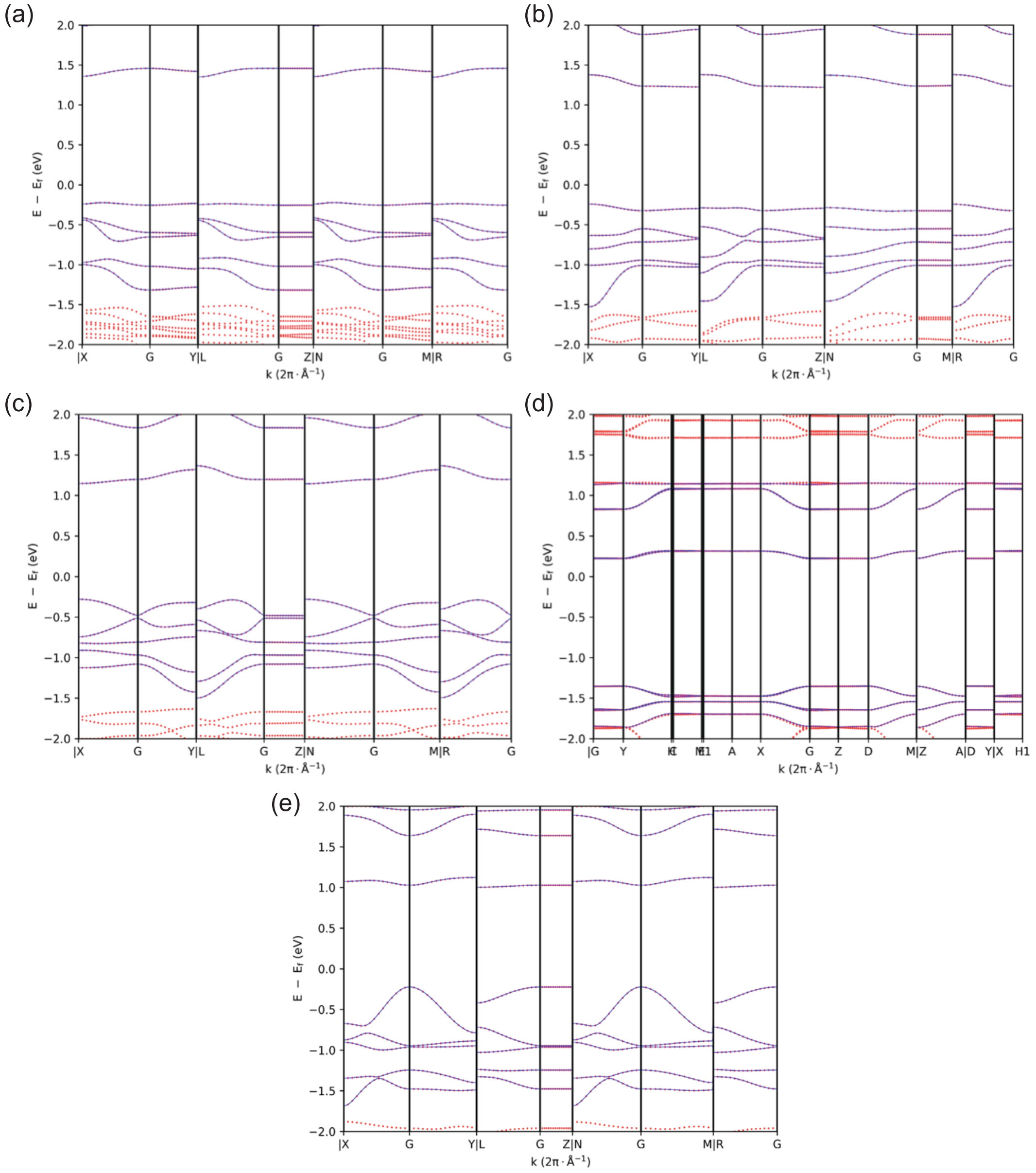

Figure S10: Electronic band structures of 3b (a), 3b-i (b), 3b-b (c), 3b-p (d), and 3b-h (e).

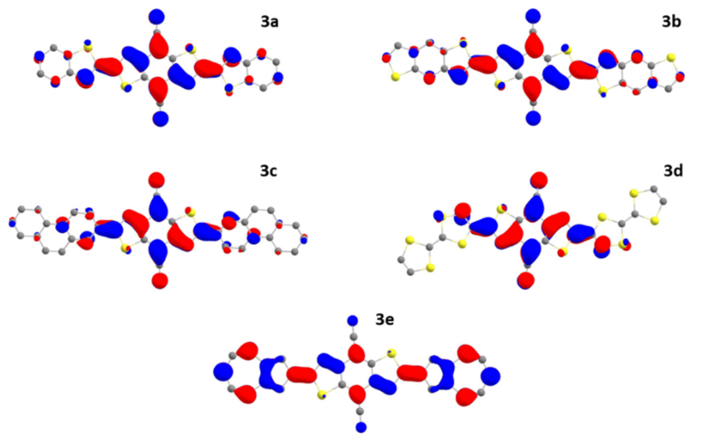

Figure S11: Pictorial representations of the lowest unoccupied molecular orbital (LUMO) of **3a-e** where the degree of LUMO delocalization is influenced by the pendant group. The side chains are trimmed down to alkynyl groups in the calculations and hydrogens are hidden for clarity purpose

V. Thin-Film Characterization

GIXD Characterization

GIXD experiments were conducted at the Cornell High Energy Synchrotron Source on the G1 station (9.95 ± 0.05 keV). The X-ray beam was aligned between the critical angles of the film and substrate, at 0.17° relative to the substrate surface. The scattered X-rays were collected on a two-dimensional CCD detector, and all GIXD images have been background subtracted. Background subtracted GIXD patterns of aligned drop-cast films were fit using DPC toolkit.[29]

Photoelectron Spectroscopies

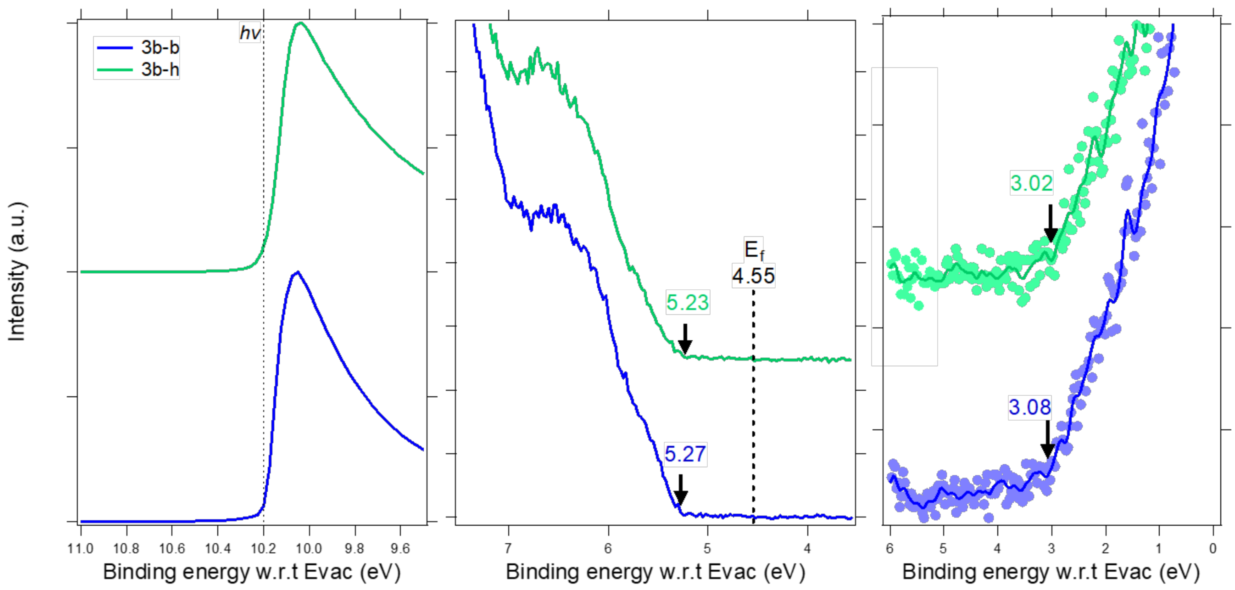

Figure S12. Ultraviolet photoelectron spectroscopy (left and center) and inverse photoelectron spectroscopy (right) of **3b-b** and **3b-h** films

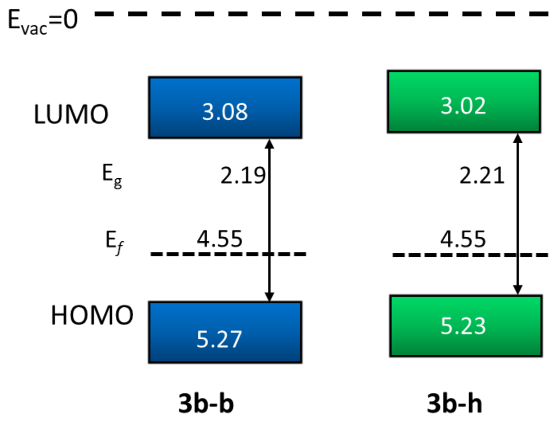

Figure S13. Interfacial energy diagrams for **3b-b** and **3b-h** films as determined from ultraviolet and inverse photoelectron spectroscopies

Thin Film Fabrication for PES

Indium tin oxide (ITO) coated substrates were used to ensure good electronic contact between the sample holder and deposited films. ITO coated glass substrates that were cleaned by sequential sonication in soap water, acetone, and isopropanol followed by UV-ozone cleaning. **3b-b** and **3b-h** films were fabricated from 5 mg/mL solutions in chloroform by spin coating at 750 rpm for 30s with 375 rpm/s acceleration.

PES Characterization

UPS measurements were conducted in a PHI 5600 UHV system with an 11 inch diameter hemispherical electron energy analyzer with a multichannel detector. The photo source for the UPS measurements was an Excitech H Lyman-a lamp (E-LUX™ 121) coupled with a 90° ellipsoidal mirror (E-LUX™ EEM Optical Module) with a dry oxygen purge of the beam path at 7.5 – 8.5 Torr, as detailed in a previous publication. All UPS measurements were recorded with -5 V sample bias and a pass energy of 5 eV. IPES measurements were performed using the bremsstrahlung isochromat mode with electron kinetic energies below 5 keV to minimize sample damage. The low energy electron beam was generated using a Kimball Physics ELG-2 electron gun equipped with a low temperature (1150 K) BaO cathode. Emitted photos were collected and focused with a fused silica bi-convex lens into the photo detector consisting of an optical bandpass filter (214 nm, Andover Corporation) and a photomultiplier tube (R585, Hamamatsu Photonics). During all IPES measurements the UHV chamber was blacked-out to exclude external light and samples were held under a -20 V bias.

References

1. M. Mamada, D. Kumaki, J. Nishida, S. Tokito, Y. Yamashita *ACS Appl. Mater. & Interf.* 2010, **2**, 1303-1307.
2. K. Takimiya, Y. Konda, H. Ebata, N. Niihara and T. Otsubo, *J. Org. Chem.*, 2005, **70**, 10569-10571.
3. S. Ito, A. Nomura, N. Morita, C. Kabuto, H. Kobayashi, S. Maejima, K. Fujimori and M. Yasunami, *J. Org. Chem.*, 2002, **67**, 7295-7302.
4. A. J. Moore and M. R. Bryce, *Synthesis*, 1997, **1997**, 407-409.

5. F. Riobe, N. Avarvari, P. Grosshans, H. Sidorenkova, T. Berclaz and M. Geoffroy, *Phys. Chem. Chem. Phys.*, 2010, **12**, 9650-9660.
6. E. Kumarasamy, S. N. Sanders, A. B. Pun, S. A. Vaselabadi, J. Z. Low, M. Y. Sfeir, M. L. Steigerwald, G. E. Stein and L. M. Campos, *Macromolecules*, 2016, **49**, 1279-1285.
7. Ł. Struk and J. G. Sośnicki, *Synthesis*, 2012, **44**, 735-746.
8. Bruker-AXS, *APEX2 Bruker-AXS Inc*, 2011.
9. Bruker-AXS, *APEX3 Bruker-AXS Inc.*, 2016.
10. L. Krause, R. Herbst-Irmer, G. M. Sheldrick and D. Stalke, *J. Appl. Crystallogr.*, 2015, **48**, 3-10.
11. G. M. Sheldrick, *Acta Crystallogr A Found Adv*, 2015, **71**, 3-8.
12. G. M. Sheldrick, *Acta Crystallogr C Struct Chem*, 2015, **71**, 3-8.
13. *International Tables for Crystallography, vol C: Mathematical, Physical and Chemical Tables*, Kluwer Academic Publishers, Holland, 1 edn., 1992.
14. S. Ito, T. Kubo, N. Morita, Y. Matsui, T. Watanabe, A. Ohta, K. Fujimori, T. Murafuji, Y. Sugihara and A. Tajiri, *Tetrahedron Lett.*, 2004, **45**, 2891-2894.
15. K. Nakayama, Y. Hirose, J. Soeda, M. Yoshizumi, T. Uemura, M. Uno, W. Li, M. J. Kang, M. Yamagishi, Y. Okada, E. Miyazaki, Y. Nakazawa, A. Nakao, K. Takimiya and J. Takeya, *Adv. Mater.*, 2011, **23**, 1626-1629.
16. W. Brutting, in *Physics of Organic Semiconductors*, ed. W. Brutting, John Wiley & Sons, 2 edn., 2006, pp. 410-412.
17. G. Kresse and J. Furthmüller, *Physical review B*, 1996, **54**, 11169.
18. G. Kresse and J. Furthmüller, *Computational Materials Science*, 1996, **6**, 15-50.
19. J. P. Perdew, J. A. Chevary, S. H. Vosko, K. A. Jackson, M. R. Pederson, D. J. Singh and C. Fiolhais, *Physical Review B*, 1992, **46**, 6671.
20. G. Kresse and D. Joubert, *Physical Review B*, 1999, **59**, 1758.
21. S. Curtarolo, G. Hart, W. Setyawan, M. Mehl, M. Jahnatek, R. Chepulskii, O. Levy and D. Morgan, *Journal*, 2009.
22. W. Setyawan and S. Curtarolo, *Computational Materials Science*, 2010, **49**, 299-312.
23. C. S. A. Fonari, *Effective Mass Calculator*, 2012.
24. F. Weigend and R. Ahlrichs, *PCCP*, 2005, **7**, 3297-3305.
25. J.-D. Chai and M. Head-Gordon, *PCCP*, 2008, **10**, 6615-6620.
26. M. Frisch, G. Trucks, H. Schlegel, G. Scuseria, M. Robb, J. Cheeseman, G. Scalmani, V. Barone, G. Petersson and H. Nakatsuji, *Wallingford CT*, 2016.
27. S. Ryno, *Transfer Integral Calculator*, 2017.
28. E. F. Valeev, V. Coropceanu, D. A. da Silva Filho, S. Salman and J.-L. Brédas, *J. Am. Chem. Soc.*, 2006, **128**, 9882-9886.
29. A. K. Hailey, A. M. Hiszpanski, D.-M. Smilgies and Y.-L. Loo, *J. Appl. Crystallogr.*, 2014, **47**, 2090-2099.
